# Audit of Seclusion Room Standards Across the Humber Teaching NHS Foundation Trust

**DOI:** 10.1192/bjo.2025.10615

**Published:** 2025-06-20

**Authors:** Kayle Jones

**Affiliations:** Humber Teaching NHS Foundation Trust, Hull, United Kingdom

## Abstract

**Aims:** To identify a set of appropriate standards against which to evaluate all seclusion rooms across Humber NHS Foundation Trust.

To identify thematic issues relating to seclusion rooms that need addressing.

To make evidence-based recommendations to improve the standard of seclusion rooms throughout the sites.

**Methods:** We arranged to attend each clinical area in person to assess the seclusion area against a defined checklist.

Considerations were made to the time and day of attendance as well as if any areas were in use. Should they be in use we determined we would assess the area at the time of seclusion review if it would not be at detriment to the patient.

If an area was used for long-term seclusion, review would be omitted.

**Results:** Of audited areas, none were 100% compliant.

Of the areas with dedicated seclusion rooms, none had an actual bed in the room, but all were equipped with mattresses.

Compliance is not at 100% for blind spots.

No clear bedding was available in a high number of rooms.

The lights and heating appear to pose some issues in several of the seclusion areas.

Four wards, Mill View Lodge, Mill View Court, Maister Lodge and Maister Court have no dedicated seclusion rooms. Despite this there have been incidents on three of those four wards, requiring a patient to be secluded before being transferred to an appropriate suite. From looking at the data for these areas, none are 100% compliant with the recommendations and as such mean that a safe and appropriate seclusion cannot be conducted.

**Conclusion:** Through this audit we highlighted that there are key concerns across all areas of the Trust in regard to the standardisation of the seclusions rooms.

Most of the identified issues were considered 'easy fixes’ that had not yet been raised as an issue and arrangements with estates could be made to rectify them.

Unfortunately we identified three areas that did not have dedicated seclusion suites but did have seclusion policies that could be operated. Patients were being secluded in general areas in these locations, leading to increased risk for staff and patients alike. Recommendations have been made to the Trust in various clinical network meetings that these clinical areas should either have a dedicated suite made available, which is compliant with the recommendations, or that the Trust is to create a new Standard Operating Procedure (SOP) detailing how a patient needing seclusion in these areas is to be managed until an appropriate seclusion suite is identified for them.

